# Performance Bound for Joint Multiple Parameter Target Estimation in Sparse Stepped-Frequency Radar: A Comparison Analysis

**DOI:** 10.3390/s19092002

**Published:** 2019-04-29

**Authors:** Qiushi Chen, Xin Zhang, Qiang Yang, Lei Ye, Mengxiao Zhao

**Affiliations:** 1 Department of Electronic and Information Engineering, Harbin Institute of Technology, Harbin 150001, China; chenqiushi@126.com (Q.C.); zhangxinhit@hit.edu.cn (X.Z.); yedaily@163.com (L.Y.); zmx_zhao@163.com (M.Z.); 2 Key Laboratory of Marine Environmental Monitoring and Information Processing, Ministry of Industry and Information Technology, Harbin 150001, China

**Keywords:** targets estimation and detection, performance analysis, sparse stepped frequency, Cramér-Rao lower bound

## Abstract

A performance bound—Cramér-Rao lower bound (CRLB) for target estimation and detection in sparse stepped frequency radars is presented. The vector formulation of this CRLB is used to obtain a lower bound on the estimation error. The estimation performance can be transformed into different types of CRLB structures. Therefore, the expressions of bounds under three equivalent models are derived separately: time delay and Doppler stretch estimator, joint multiple parameter estimator, and sparse-based estimator. The variables to be estimated include the variances of unknown noise, range, velocity, and the real and imaginary parts of the amplitude. A general performance expression is proposed by considering the echo of the target in the line-of-sight. When the relationship between CRLB and various parameters are discussed in detail, the specific effect of waveform parameters on a single CRLB is compared and analyzed. Numerical simulations demonstrated that the resulting CRLB exhibits considerable theoretical and practical significance for the selection of optimal waveform parameters.

## 1. Introduction

A sparse stepped frequency (SSF) signal extends the traditional continuous bandwidth to random discontinuous frequency bands in modern radars. However, the performance of SSF signals has not been evaluated effectively. The Cramér-Rao lower bound (CRLB) expresses the lower bound of the variance of unbiased estimators in [1,2], which has a wide range of applications in radar. Various CRLBs of joint parameter estimation for broadband signals have been proposed.

In [2], a Gaussian signal and a linear frequency modulation (LFM) signal are analyzed. It is verified that the CRLB of joint estimation depends on the signal-noise-ratio (SNR), threshold, and signal structure. In [3,4], an LFM signal is used to analyze the CRLB of joint Range–Doppler(RD) estimation performance in both active radar and distributed passive radar networks. Particularly in [5], the CRLB of RD estimation is computed using FM commercial radio signals for passive radar network systems; this demonstrates that the coherent CRLB is much lower than that of the noncoherent processing mode. Subsequently, a modified CRLB is investigated in [6]; it is applicable for passive multistatic radar systems with antenna arrays. Analysis confirmed that the joint estimation performance is related to the geometry of the target, radar configuration, SNR, and signal parameters. Reference [7] investigates the CRLB of the joint target radial velocity and the acceleration estimation performance of an linear stepped frequency (LSF) signal. In [8], the CRLB expression is derived to guide TDOA estimation using a frequency-hopping signal. Similarly, in [9,10], the performance of joint TD estimation is studied with random stepped frequency signals. In addition, some researchers have explored the performances of other parameters; e.g., in [11,12], the CRLB of a target’s position, intensity, and geometry type are derived by considering the signal as a geometric theory of diffraction (GTD)-based scattering center model. The CRLBs of a known and an unknown phase for joint RD estimation is presented in [13]; the unknown time delay, Doppler stretch, amplitude, and uniform distributed initial phase are estimated simultaneously in [14]. Although the target estimation of an SSF signal has been employed in wideband radar system [15,16,17], the CRLB performance expression above for various waveforms cannot be applied directly to an SSF. In fact, compared with an LFM or LSF signal with limited continuous bandwidth, an SSF exhibits a higher synthetic bandwidth, thus, enabling it to improve the resolution of separating multiple close-range spatial targets. Moreover, because its carrier frequency is sparse and exhibits random hopping, the range–Doppler coupling problem can be suppressed effectively [18]; further, the interference from other users can be avoided.

The parameters of target echo, and environmental and transmit waveforms affect the performance significantly. Therefore, the relationship among these parameters and the estimation performance are compared and analyzed. Some conclusions have been drawn from the following studies. In [19], the CRB for the unbiased estimators of parameters from compressed samples are investigated. The CRB increases when the number of compressed samples is larger than that of the targets. In [20], the CRLB criterion of time delay estimation is analyzed under various pulse shapes. It is concluded that the performance depends on the pulse’s period, order, and shape. In [21], the estimation performances for uniform and nonuniform frequency samplings are analyzed. The best sampling set is gained by statistical strategy. In [2], it verifies that the CRLB of the joint estimation depends on the SNR, threshold, and signal structure. In [14], the performances of unknown time delay, Doppler stretch, amplitude, and uniform distributed initial phase are explored simultaneously. In [22,23], primary factors including the SNR, central carrier frequency, carrier frequency bandwidths and frequency shifting code words that affect the performance are discussed.

These expressions effectively explain the CRLB for target estimation in previous references. However, because of the sparseness and irregularity of the selected frequencies, the existing regularity for linear frequencies is no longer applicable in the sparse signal. Therefore, a new process of performance analysis for an SSF signal is necessary. In this work, the estimation problem using an SSF signal as a transmitting waveform can be equivalent to that of the three different structures. The accordance expression of a special CRLB deduction can be obtained based on a variant SSF. All relevant variables are retained without simplification in the process to facilitate the further analysis of the parameter relationships accordingly.

The primary work and contributions can be stated as follows: First, by referring to the CRLB of delay and Doppler stretch equivalent model used in the wideband signal [2] and in random stepped frequency signal [9], a general representation of the same performance is derived by considering an SSF signal with a chirp envelope. After setting some of the parameters, the CRLBs of an SSF signal with a rectangular envelope are verified. Next, according to the GTD-based scattering center estimation [11], the similar CRLB for multiple parameter estimations using an SSF waveform is derived. The actual performances under various model assumptions are analyzed comprehensively and systematically. Subsequently, a sparse-based expression for model error [24] is substituted directly into the SSF estimation for introducing the CS estimation method. Finally, the degree and trend of each individual coefficient constituting the bound of the entire joint estimation are discussed. The relationship between the CRLB and various parameters are developed in detail. The resulting CRLB above presents instructional significance for transmitting waveform optimization.

The remainder of this paper is organized as follows. In Section 2, the signal model for the SSF signal is introduced. In Section 3, The CRLB of basic LSF and SSF signals within a time delay and Doppler stretch model are presented. In Section 4, the CRLB of joint multiple parameter estimation is discussed. In Section 5, the CRLB of a sparse-based estimator is introduced. Numerical simulation examples and results are described in Section 6 and conclusions are presented.

**Notation** **1.** A capital bold letter x represents a matrix. $x\left(n\right)$ is the $n^{th}$ sample point. A letter with superscript $\hat{\theta }$ represents the estimation of θ. $E\left\{\;\;\cdot \;\;\right\}$ represents the expectation operator. $\mathrm{Tr}\left(\;\;\cdot \;\right)$ denotes the trace of matrix. $\left\lfloor \;\;\cdot \;\right\rfloor$ is the rounding operator. ${∥\;\;\;\cdot \;\;\;∥}_{0}$ represents the $\ell_{0}$ norm. ${∥\;\;\;\cdot \;\;\;∥}_{2}$ is the standard Euclidean norm with the value ${∥\;\;x\;∥}_{2}=\sqrt{\sum_{i}{\left|x_{i}\right|}^{2}}$. The symbol ∂ is the partial derivative operator. ${\left(\;\;\cdot \;\right)}^{*}$, ${\left(\;\;\cdot \;\right)}^{T}$, and ${\left(\;\;\cdot \;\right)}^{H}$ indicate the conjugate, transpose, and conjugate transpose.

## 2. Signal Model

In this study, $t_{n}$ denotes the sampling time of the $n^{th}$ pulse; the width of every narrow band pulse is $T_{w}$, pulse repetition interval (PRI) is *T*. Therefore, the transmitting signal is

(1) $$x_{0}\left(t\right)=\sum_{n=0}^{N-1}\mathrm{rect}\left(\frac{t-nT}{T_{w}}\right)e^{-j2\pi f_{n}t}$$

where rect(·) stands for a rectangular function with

$$\mathrm{rect}\left(\frac{t}{T}\right)=\left\{\begin{array}{c} 1\;\;\;\;,\;\;\;\;\;-T/2\leq t\leq T/2\\ 0\;\;\;,\;\;\;\;\;\mathrm{else} \end{array}\right.$$

$f_{n}$ is set as the chosen frequency for every pulse. It is noteworthy that the transmitting frequencies can be calculated by $f_{n}=f_{0}+C_{n}\Delta f$, where $f_{0}$ is the initial carrier frequency, and Δf is the frequency step. The sequence of frequencies is $C_{n}\in \left[1,M\right]$ with $N={∥C∥}_{0}<={∥m∥}_{0}=M$, where *M* is the total multiples of step Δf in the whole frequency span, and *N* is the number of transmitting pulses. The frequency pattern is determined by *n* and $C_{n}$.

The receiving signal is the time delay and Doppler stretch version of the transmitting signal. After processing by mixing and sampling, the discrete form of the echo is

(2) $$\begin{array}{cc} y(n) & =x(n)+w(n)\\ & =\sum_{k=1}^{K}A_{k}e^{-j4\pi \left(f_{0}+C_{n}\Delta f\right)\left(R_{k}+V_{k}nT\right)/c}\;+w\;\left(n\right)\; \end{array}$$

where the $k^{th}$ target signal consists of the range $R_{k}$, velocity $V_{k}$, and echo amplitude $A_{k}$. When K=1, x(n) in (2) can be simplified as

(3) $$x\left(n\right)=Ae^{-j4\pi f_{n}\left(R+VnT\right)/c}\;.$$

Defining $y={\left[y\left(1\right),y\left(2\right),\ldots ,y\left(n\right)\right]}^{T}$ and $x={\left[x\left(1\right),x\left(2\right),\ldots ,x\left(n\right)\right]}^{T}$, the matrix form is given by y=x+w. w(n) is the zero-mean white Gaussian noise (WGN), which is distributed as $w∼N(0,\sigma^{2})$. The estimation performance for this type of signal model will be studied in the next section.

## 3. CRLB of Basic LSF and SSF Signals—Time Delay And Doppler Stretch

We derived a performance bound for the SSF waveform. The relationship among all the parameters is analyzed to provide useful information for waveform optimization. From this theory performance, the range–Doppler information can be estimated more efficiently in the radar system.

The receiving signal can be transferred into the time delay and Doppler stretch versions as the form in [2] from the transmitting signal, that is

(4) $$s\left(t\right)=ax\left(\left(t-\tau \right)/\sigma \right)+w\left(t\right)$$

where τ=2R/c is the time delay of the echo from the target at distance *R* (range is proportional to time delay; therefore, we use the time delay to explain the estimation in the range domain), *c* is the speed of light, $\sigma =\frac{c+v}{c-v}$ is the Doppler stretch, *a* is the amplitude. Here, we define the SNR using the signal power *E* and power spectrum density (PSD) $N_{0}$ of w(t), that is

(5) $$E=a^{2}\int_{-\infty }^{\infty }{\left|s(t)\right|}^{2}dt.$$

We will consider the estimation performance for this type of signal model. The CRLBs of the joint time and Doppler estimation are derived. The probability density function(PDF) p(s|θ) of the complex echo signal *x* is given by

(6) $$p\left(s|\theta \right)=K\exp\left\{-\frac{1}{N_{0}}\int_{-\infty }^{\infty }{\left|s\left(t\right)-ax\left(\sigma \left(t-\tau \right)\right)\right|}^{2}dt\right\}$$

where *K* is a constant, and θ=[τσ] is the parameter vector to be estimated. The covariance matrix for an unbiased estimate $\hat{\theta }$ satisfies

(7) $$\mathrm{CRL}B_{\hat{\theta }}=E\left[(\hat{\theta }-\theta )\cdot {\left(\hat{\theta }-\theta \right)}^{T}\right]\geq J^{-1}$$

where ${\left(\right)}^{T}$ is the transpose of a vector, and J is the Fisher information matrix (FIM), which is defined by [1,2]

(8) $$J\left(\theta \right)=E\left\{\nabla_{\theta }\ln p\left(s|\theta \right)\cdot {\left[\nabla_{\theta }\ln p\left(s|\theta \right)\right]}^{T}\right\}$$

Therefore, the FIM is

(9) $$J^{-1}\left(\theta \right)=\frac{N_{0}\sigma }{2{\left|a\right|}^{2}\left(D_{s}B_{s}-{C_{s}}^{2}\right)}\left[\begin{array}{cc} D_{s} & -C_{s}\\ -C_{s} & B_{s} \end{array}\right]$$

where $B_{s}\overset{\Delta }{\;=}\int_{-\infty }^{\infty }{\left|\dot{s}\left(t\right)\right|}^{2}dt$, $C_{s}\overset{\Delta }{\;=}\int_{-\infty }^{\infty }t{\left|\dot{s}\left(t\right)\right|}^{2}dt$, $D_{s}\overset{\Delta }{\;=}\int_{-\infty }^{\infty }t^{2}{\left|\dot{s}\left(t\right)\right|}^{2}dt$, $\dot{s}\left(t\right)=ds\left(t\right)/dt$. The CRLBs of the time delay and Doppler stretch can be specifically expressed as

(10) $$\mathrm{var}\left(\hat{\tau }\right)={\left[J^{-1}\left(\theta \right)\right]}_{11}=\frac{N_{0}\sigma D_{s}}{2{\left|a\right|}^{2}\left(D_{s}B_{s}-{C_{s}}^{2}\right)}$$

(11) $$\mathrm{var}\left(\hat{\sigma }\right)={\left[J^{-1}\left(\theta \right)\right]}_{22}=\frac{N_{0}\sigma B_{s}}{2{\left|a\right|}^{2}\left(D_{s}B_{s}-{C_{s}}^{2}\right)}$$

### 3.1. CRLB of SSF-Chirp Signals

From (10) and (11), the CRLB performance of the signal waveform depends on the signal parameters $B_{s}$, $C_{s}$, and $D_{s}$. We impose SF signal (4) into them, and subsequently calculate the theoretical performances of the CRLBs. This rectangular function can be expressed in the form of a unit step function, that is

(12) $$\int_{-\infty }^{+\infty }s\left(t\right)\left[u\left(t+\frac{T_{w}}{2}\right)-u\left(t-\frac{T_{w}}{2}\right)\right]dt=\int_{nT-\frac{T_{w}}{2}}^{nT+\frac{T_{w}}{2}}s(t)dt.$$

Therefore, the first power of $t_{n}$ can be calculated under the interval from $\left(nT-\frac{T_{w}}{2}\right)$ to $\left(nT+\frac{T_{w}}{2}\right)$; therefore, $t_{n}\left|{}_{nT-\frac{T_{w}}{2}}^{nT+\frac{T_{w}}{2}}\right.=T_{w}$ is obtained. Similarly, the quadratic term is $t_{n}^{2}\left|{}_{nT-\frac{T_{w}}{2}}^{nT+\frac{T_{w}}{2}}\right.=2nTT_{w}$, the third term is $t_{n}^{3}\left|{}_{nT-\frac{T_{w}}{2}}^{nT+\frac{T_{w}}{2}}\right.=\frac{T_{w}^{3}}{4}+3n^{2}T_{w}T^{2}$, the forth term is $t_{n}^{4}\left|{}_{nT-\frac{T_{w}}{2}}^{nT+\frac{T_{w}}{2}}\right.=T_{w}^{3}Tn+4n^{3}T_{w}T^{3}$ and the fifth term is $t_{n}^{5}\left|{}_{nT-\frac{T_{w}}{2}}^{nT+\frac{T_{w}}{2}}\right.=\frac{T_{w}^{5}}{16}+\frac{5n^{2}T_{w}^{3}T^{2}}{2}+5n^{4}T_{w}T^{4}$. To simplify the expressions above, we introduce $Z_{\left(i,j\right)}=\sum_{n=0}^{N-1}n^{i}{f_{n}}^{j}$(0≤i≤4,0≤j≤2) into the following deviation. The parameter values of a chirp envelope are as follows:

(13) $$\left\{\begin{array}{c} \;\left(B_{s}\right){}_{chirp}=4\pi^{2}\left(\begin{array}{c} z_{\left(0,2\right)}+\gamma Tz_{\left(1,1\right)}\\ +\frac{1}{4}\gamma^{2}T^{2}z_{\left(2,0\right)}+\frac{1}{48}\gamma^{2}NT_{w}^{2} \end{array}\right)\\ \;{\left(C_{s}\right)}_{chirp}=4\pi^{2}\left(\begin{array}{c} \frac{\gamma T_{w}^{2}}{12}z_{\left(0,1\right)}+\frac{1}{16}\gamma^{2}T_{w}^{2}Tz_{\left(1,0\right)}+Tz_{\left(1,2\right)}\\ +\gamma T^{2}z_{\left(2,1\right)}+\frac{1}{4}\gamma^{2}T^{3}z_{\left(3,0\right)} \end{array}\right)\\ \;{\left(D_{s}\right)}_{chirp}=4\pi^{2}\left(\begin{array}{c} \frac{T_{w}^{2}}{12}Z_{\left(0,2\right)}+\frac{1}{4}\gamma T_{w}^{2}Tz_{\left(1,1\right)}\\ +\frac{\gamma^{2}T_{w}^{2}T^{2}}{8}z_{\left(2,0\right)}+T^{2}Z_{\left(2,2\right)}\\ +\gamma T^{3}z_{\left(3,1\right)}+\frac{\gamma^{2}T^{4}}{4}z_{\left(4,0\right)}+\frac{\gamma^{2}T_{w}^{4}N}{320} \end{array}\right) \end{array}\right.$$

The values of the CRLBs depend on not only the total number of pulses *M*, but also the selected frequencies $f_{n}\;\;$, but the frequency pattern from the relationship between sequence of pulses *n* and sequence of frequency $C_{n}$.

### 3.2. SSF-Rect Signals

When the slope with the value γ=0, the envelope of the SSF signal changes from chirp to rectangular, namely an SSF-rect signal. Therefore, substituting γ=0 into (13), the intermediate variables for an SSF-rect signal are

(14) $$\left\{\begin{array}{c} \left(B_{s}\right){\;}_{rect}\;=4\pi^{2}T_{w}z_{\left(0,2\right)}\\ \left(C_{s}\;\right){\;}_{rect}\;=4\pi^{2}TT_{w}z_{\left(1,2\right)}\\ {\left(D_{s}\;\right)}_{rect}=4\pi^{2}T_{w}\left(T^{2}z_{\left(2,2\right)}+T_{w}^{2}z_{\left(0,2\right)}/12\right) \end{array}\right.$$

When N=M, $C_{n}=n$, the signal will be in the form of an LSF signal. The expression is the same as that of the SSF for cases involving a chirp envelope and a rectangular envelope. Similarly, when n=0, N=1, $f_{n}=f$, SSF signal will degenerate into LFM signal. We obtain $z_{\left(0,1\right)}=f_{0}$, other values in $z_{\left(i,j\right)}$ are equal to 0.

## 4. CRLB of Joint Multiple Parameter Estimation

### 4.1. Basic Model of CRLB

In this section, the CRLBs of the joint multiple parameter estimation are derived. The PDF of the complex echo signal *y* is given by

(15) $$\begin{array}{cc} p\left(y|\theta \right) & ={\left(\frac{1}{\pi \sigma^{2}}\right)}^{N}\exp\left\{-\sum_{n=1}^{N}\frac{{\left|y(n)-x(n)\right|}^{2}}{\sigma^{2}}\right\}\\ & =\prod_{n=1}^{N}\frac{1}{\pi \sigma^{2}}\exp\left\{-\frac{{\left|y(n)-x(n)\right|}^{2}}{\sigma^{2}}\right\} \end{array}$$

where θ is the parameter vector to be estimated. The variables in the definition $\theta =[\sigma^{2}|R|V|A_{r}|A_{i}]$ represent the variance of unknown noise, range, velocity, and real and imaginary parts of the amplitude, correspondingly. According to [1,2], the CRLB matrix for an unbiased estimate $\hat{\theta }$ is the same as that in (7) and (8). We have

(16) $$\nabla_{\theta }\ln p(x|\theta )={\left[\frac{\partial }{\partial \sigma^{2}},\frac{\partial }{\partial R},\frac{\partial }{\partial V},\frac{\partial }{\partial A_{r}},\frac{\partial }{\partial A_{i}}\right]}^{T}$$

Therefore, each item in the FIM can be calculated by

(17) $$J_{ij}=E\left[\frac{\partial \ln p(y|\theta )}{\partial \theta_{i}}\frac{\partial \ln p{\left(y|\theta \right)}^{H}}{\partial \theta_{j}}\right]$$

### 4.2. Series Expressions of Partial Derivative

The log-likelihood function can be obtained by taking the natural logarithm of (15)

(18) $$\begin{array}{cc} \ln p(y|\theta ) & =\ln{\left(\pi \right)}^{-N}+\ln{\left(\sigma^{2}\right)}^{-N}+\left\{-\frac{1}{\sigma^{2}}\sum_{n=1}^{N}{\left|y(n)-x(n)\right|}^{2}\right\}\\ & =-N\ln\pi -N\ln\sigma^{2}-\frac{1}{\sigma^{2}}\left[{\left(y-x\right)}^{H}\left(y-x\right)\right] \end{array}$$

The partial derivative for the noise variance is derived:

(19) $$\frac{\partial \ln p(y|\theta )}{\partial \sigma^{2}}=-\frac{N}{\sigma^{2}}+\frac{1}{\sigma^{4}}\left[{\left(y-x\right)}^{H}\left(y-x\right)\right]$$

Subsequently, the derivation for range is calculated as follows:

(20) $$\frac{\partial \ln p(y|\theta )}{\partial R}=-\frac{1}{\sigma^{2}}\frac{\partial \left[{\left(y-x\right)}^{H}\left(y-x\right)\right]}{\partial R}$$

According to $\frac{\partial x}{\partial R}=\frac{-j4\pi }{c}\mathrm{Fx}$, the partial derivation equation above can be written as

(21) ∂lnp(y|θ)∂R=−8πσ2cRejFxHw

Similarly, the partial derivative for the velocity term is $\frac{\partial x}{\partial V}=\frac{-j4\pi T}{c}\mathrm{nFx}$.

(22) ∂lnp(y|θ)∂V=−8πTσ2cRejnFxHw

For the real and imaginary parts of the amplitude, let $A=A_{r}+A_{i}$, the corresponding derivations are found respectively as follows

(23) ∂lnp(y|θ)∂Ar=−2σ2Re1AHxHw

(24) ∂lnp(y|θ)∂Ai=2σ2Re1AHjxHw

From the deduction of the above, the integrated matrix can be expressed as

(25) ∇θlnp(x|θ)=−Nσ2+1σ4wHw−8πσ2cRejFxHw−8πTσ2cRejnFxHw−2σ2Re1AHxHw2σ2Re1AHjxHw

### 4.3. Derivations of the FIM

The FIM elements are calculated in this section. (17) is split into the combinatorial form

(26) $$J_{\sigma^{2}RVA_{r}A_{i}}=\left[\begin{array}{cc} J_{\sigma^{2}} & 0\\ 0 & \begin{array}{cc} J_{RV} & J_{\bar{RV}\bar{A_{r}A_{i}}}\\ J_{\bar{A_{r}A_{i}}\bar{RV}} & J_{A_{r}A_{i}} \end{array} \end{array}\right]$$

Because the measurement noise w obeys the complex normal distribution, some important properties are satisfied, as follows

(27) $$\begin{array}{c} E\left\{w\right\}=0_{N\times 1}\\ E\left\{ww^{T}\right\}=0_{N\times N}\\ E\left\{ww^{H}\right\}=\sigma^{2}I_{N\times N}\\ E\left\{w^{H}w\right\}=N\sigma^{2}\\ E\left\{w^{H}ww^{H}w\right\}=N\left(N+1\right)\sigma^{4}\\ E\left\{w^{*}w^{T}\right\}=E\left\{{\left(ww^{H}\right)}^{T}\right\}=\sigma^{2}\\ E\left\{w^{*}w^{H}\right\}=E\left\{{\left(ww^{T}\right)}^{*}\right\}=0_{N\times N} \end{array}$$

Considering the variance of noise as a constant related to the distribution, the first item in (26) can be extracted for calculation. According to these properties, $J_{\sigma^{2}}$ can be calculated by

(28) $$\begin{array}{c} J_{\sigma^{2}}=\sigma^{-4}E\left[\frac{\partial \ln p(y|\theta )}{\partial (\sigma^{2})}{\frac{\partial \ln p(y|\theta )}{\partial (\sigma^{2})}}^{H}\right]\\ =\sigma^{-4}E\left[N^{2}-2N\sigma^{-2}w^{H}w+\sigma^{-4}w^{H}ww^{H}w\right]\\ =\sigma^{-4}N \end{array}$$

A useful formula can be extracted to reduce the computational complexity. For two arbitrary matrices P and Q, the expectation after multiplying the real part of two matrices satisfies the following rule

(29) ERePwReQwH=14EPw+P*w*wHQH+wTQT=σ22Re(PQH)

Additionally, Γ=jFx is set to simplify the expression. It is apparent that ${\left(j\mathrm{nFx}\right)}^{H}=\Gamma^{H}n$, where n is a real number diagonal matrix with $n=n^{H}=n^{T}$. Substituting this symbol into $J_{RV}$ and $J_{A_{r}A_{i}}$ and calculating the expectation of the unbiased estimation error, every item in (26) can be simplified using this rule, which are

(30) EReΓHwReΓHwH=12σ2Re(ΓHΓ)EReΓHwReΓHnwH=12σ2Re(ΓHnΓ)EReΓHnwReΓHwH=12σ2Re(ΓHnΓ)EReΓHnwReΓHnwH=12σ2ReΓHn2ΓERe1AHxHwRewHx1A=σ22Re1AAHxHxE−Re1AHxHwRewHjx1A=σ22Im1AAHxHxE−Re1AHjxHwRewHx1A=−σ22Im1AAHxHxERe1AHjxHwRewHjx1A=σ22Re1AAHxHx

To integrate the processes above, the submatrices can be written as follows.

(31) JRV=32π2σ2c2Re(ΓHΓ)TRe(ΓHnΓ)TRe(ΓHnΓ)T2ReΓHn2Γ

(32) JArAi=σ22Re1AAHxHxIm1AAHxHx−Im1AAHxHxRe1AAHxHx

The remaining eight items in J can be obtained separately, which are

(33) E(R,Ar)=E∂lnp(y|θ)∂(R)∂lnp(y|θ)∂(Ar)H=E−8πσ2cReΓHw−2σ2Re1AHxHwH=16πσ4cσ22Re1AΓHx=8πσ2cIm(1AxHFHx)

The similar results are given by

(34) E(R,Ai)=E∂lnp(y|θ)∂(R)∂lnp(y|θ)∂(Ai)H=−8πσ2cRe(1AxHFHx)E(V,Ar)=E∂lnp(y|θ)∂(V)∂lnp(y|θ)∂(Ai)H=8πTσ2cIm1AxHFHnHxE(V,Ai)=E∂lnp(y|θ)∂(V)∂lnp(y|θ)∂(Ai)H=−8πTσ2cRe1AxHFHnHxE(Ar,R)=E∂lnp(y|θ)∂(Ar)∂lnp(y|θ)∂(R)H=−8πσ2cIm1AHxHFxE(Ar,V)=E∂lnp(y|θ)∂(Ar)∂lnp(y|θ)∂(V)H=−8πTσ2cIm1AHxHnFxE(Ai,R)=E∂lnp(y|θ)∂(Ai)∂lnp(y|θ)∂(R)H=−8πσ2cRe1AHxHFxE(Ai,V)=E∂lnp(y|θ)∂(Ai)∂lnp(y|θ)∂(R)H=−8πTσ2cRe1AHxHnFx

Another two submatrices are summarized as

(35) JRV¯ArAi¯=8πσ2cIm(1AxHFHx)−Re(1AxHFHx)TIm1AxHFHnHx−TRe1AxHFHnHx

(36) JArAi¯RV¯=−8πσ2cIm1AHxHFxTIm1AHxHnFxRe1AHxHFxTRe1AHxHnFx

Based upon the previous derivation, the complete FIM is presented in (37) by substituting the results of the submatrices into the original (26). Subsequently, the values of the CRLBs, which characterize the estimated performance, can be calculated by substituting this FIM into (7).

(37) J=σ−4N00032π2σ2c2Re(xHFHFx)32π2σ2c2TRe(xHFHnFx)32π2σ2c2TRe(xHFHnFx)32π2σ2c2T2RexHFHn2Fx8πσ2cIm1AxHFHx−8πσ2cRe(1AxHFHx)8πTσ2cIm1AxHFHnHx−8πTσ2cRe1AxHFHnHx0−8πσ2cIm1AHxHFx−8πTσ2cIm1AHxHnFx−8πσ2cRe1AHxHFx−8πTσ2cRe1AHxHnFxσ22Re1AAHxHxσ22Im1AAHxHx−σ22Im1AAHxHxσ22Re1AAHxHx

## 5. CRLB of Sparse Based Estimator—Compressive Sensing

Compressive sensing is applicable to the sparse target recovery model for this type of undersampled signal. Unlike the performance analysis above, this method presents its own evaluation criteria when calculating the estimation performance. In this section, the CRLB performance for a CS with an SSF signal is analyzed.

In the receiving signal (3), the spaces of the range and velocity can be divided into P×Q grids. The dimensions of the range are $\left[R_{1},R_{2},\cdots ,R_{P}\right]$. Similarly, the dimensions of the velocity are $\left[V_{1},V_{2},\cdots ,V_{Q}\right]$. Therefore, the range unit can be written as ΔR=c/(2ΔfP), and the velocity unit can be written as $\Delta V=c/(2f_{0}TQ)$. At every grid, the target exhibits the corresponding scattering intensity, that is

(38) $$x\left(p,q\right)=\left\{\begin{array}{c} A,\mathrm{The}\;\;\;\;{\left(\left(p-1\right)Q+q\right)}^{th}\mathrm{grid}\;\mathrm{exists}\;a\;\mathrm{target}\\ 0,\mathrm{The}\;\mathrm{target}\;\mathrm{is}\;\mathrm{not}\;\mathrm{located}\;\mathrm{at}\;{\left(\left(p-1\right)Q+q\right)}^{th}\;\mathrm{grid} \end{array}\right.$$

The phase of the echo signal is redefined as

(39) $$\Phi_{i}\left(m,\left(p-1\right)Q+q\right)=\exp\left(j4\pi \left(f_{0}+m\Delta f\right)\left(R_{p}+V_{q}mT\right)/c\right),$$

where m∈[1,2,…,M],p∈[1,2,…,P],q∈[1,2,…,Q], and every $\Phi_{i}$ is an M×1 column vector representing the phase shift on *M* pulses caused by the target at the position of the $i^{th}$ grid. Therefore, Φ is the combination of all possible phase shifts on range velocity grids. Researchers in [24,25] have deduced the CRLB for estimating the sparse parameter. Additionally, we can obtain the performance representation from the FIM. Similar to the same concept of (7) and (8), we have

(40) $$J_{ij}=\frac{1}{\sigma^{2}}{\left(\Phi_{I}^{H}\Phi_{I}\right)}_{ij}$$

where I is the index set defined in the columns of submatrix $\Phi_{I}$ from Φ. Therefore, the unbiased estimation by the CRLB is

(41) $$E\left\{{∥\hat{x}-x∥}^{2}\right\}\geq Tr\left(J^{-1}\right)=\sigma^{2}\mathrm{Tr}\left({\left(\Phi_{I}^{H}\Phi_{I}\right)}^{-1}\right)$$

The performance characterization is related to only Φ. This rule is appropriate for the sparse target estimation. However, it is noticeable that, after compressive sensing reconstruction, the recovered results are subsequently mapped inversely to the corresponding real range velocity unit as the subscript. The largest *K* values are transformed into $\hat{x}$. If the subscript of the maximum value is located at one position ${\hat{x}}_{k}$ in all scopes PQ, the real range and velocity estimations are determined by calculation. Specifically, $R_{k}=\Delta R*\beta$, $V_{k}=\;\mathrm{mod}\;\left({\hat{x}}_{k},\beta \right)$, where $\beta =\left\lfloor {\hat{x}}_{k}/Q\right\rfloor$, $\left\lfloor \cdot \right\rfloor$ is the rounding operator, mod(a,b) denotes the remaining operators from *a* to *b*. The sparse targets can subsequently be reconstructed accurately.

## 6. Experiments and Discussion

This section introduces several simulations to examine how the CRLB performs when the parameters are changed. The discussions above are compared herein.

### 6.1. CRLB Comparison with Different Waveforms

**Figure 1:**
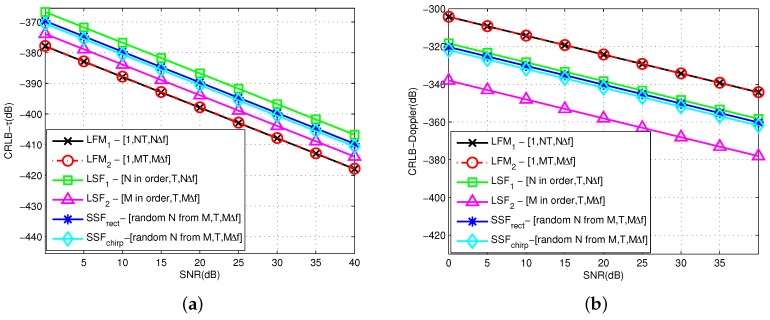
Comparison of CRLBs with different waveform. (**a**) CRLB of time delay; (**b**) CRLB of Doppler-stretch.

According to the deviation of the time delay and Doppler stretch model, we generate the CRLB by various waveforms in different parameter configurations for comparison. These waveforms include LFM${}_{1}$ and LFM${}_{2}$ with changing pulse width and total bandwidth, LSF${}_{1}$ and LSF${}_{2}$ with changing pulse number and total bandwidth, two SSF waveforms with changing envelope, namely SSF${}_{\mathrm{chirp}}$ (13) and SSF${}_{\mathrm{rect}}$ (14). In each trial, the value of SNR is set from 0 to 40dB. Other basic parameters are $T_{w}=1\;\mathrm{ms}$, T=2ms, M=100, N=50, Δf=50kHz, $f_{0}=10\;\mathrm{MHz}$. The theoretical CRLB for time delay and Doppler stretch estimation are respectively illustrated in Figure 1a,b. For the legends [A,B,C] in the figure, three parts represent the total number of pulses in a burst, pulse width for each pulse, and equivalent coverage bandwidth. “*M* in order” represents the using frequencies from 0 to M−1 multiples of Δf in sequence. “Random *N* from *M*” means *N* frequencies combination is generated as the rule of SSF from frequency span MΔf.

As shown in the figure, the performance trend of the estimation from multiple waveforms is consistent along with the increasing SNR under the same basic parameters. However, the difference exists between time estimation and Doppler estimation. For time delay estimation, the CRLB of SF-based waveforms are obviously not as good as that of LFM signal, but have better performance in Doppler estimation. When the bandwidth range and pulse width change, it has slight influence on the estimation for LFM. With the increasing bandwidth in LSF, the performance improves significantly. By comparison, the CRLB of SSF with *N* sparse frequencies is used between *N* pulses and *M* pulses in LSF signal. Moreover, the envelope plays an insignificant role.

### 6.2. Comparison of Different CRLBs

For the deviations in the time delay and Doppler stretch model, we use the CRLB of SSF-chirp signal as an example. The obtained statistical rules are also applied to the SSF-rect and LSF signals based on a reasonable definition of variables.

**Figure 2:**
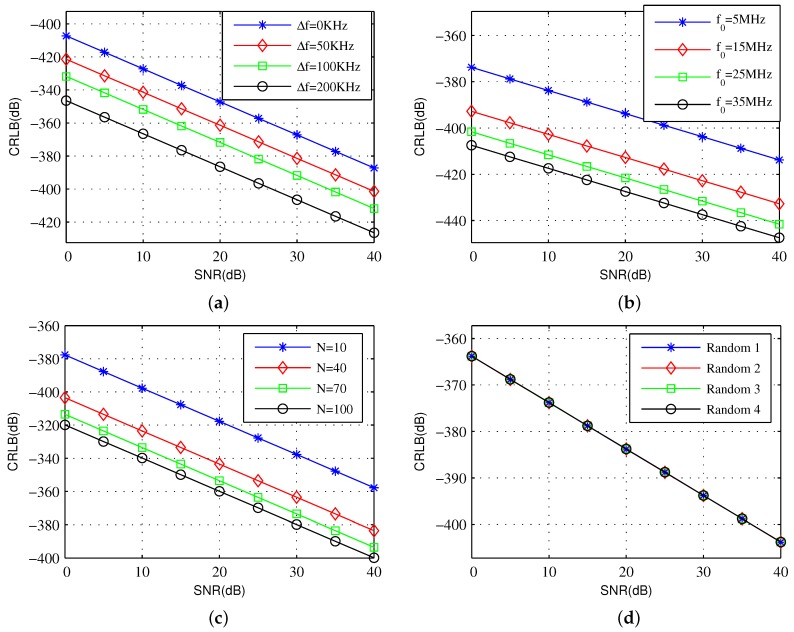
CRLB comparison with different parameters. (**a**) different frequency step; (**b**) different initial carrier frequency; (**c**) different frequency number; (**d**) different sparse frequency combination.

For each SNR value from 0 to 40 dB, we calculate the theoretical $\mathrm{var}{\left(\hat{\tau }\right)}_{ship}$ of 500 independent Monte Carlo simulations. In each trial, the frequency combination is generated randomly from the frequency span. The basic parameters are $T_{w}=1\;\mathrm{ms}$, T=5ms, M=100, N=50, Δf=1KHz, $f_{0}=5\;\mathrm{MHz}$. The estimation performances with different frequency steps are illustrated in Figure 2a. The basic parameters are set as the above setting while Δf is set to 0 kHz, 50 kHz, 100 kHz, and 200 kHz for comparison. Similarly, $f_{0}$ is set to 5 MHz, 15 MHz, 25 MHz and 35 MHz in Figure 2b; frequency number *N* is set to 10, 40, 70, and 100 in Figure 2c. Any four groups of sparse frequency combination are selected from all the tests to compare in Figure 2d.

As shown in the figure, the changes in Δf, $f_{0}$, and *N* exhibit corresponding effects on the range of the final CRLB. However, only Δf exhibits uniform variations, the other two variables exhibit differentiations under equidistant values. The effect of random combination is minimal when the other conditions remain unchanged.

The CRLB of the joint multiple parameter estimation is the same as in (4). Every normalized ${\mathrm{CRLB}}_{i}$ as the $i^{th}$ independent parameter can also be calculated individually. For every SNR value from 0 to 50dB, the theoretical CRLB is calculated. In each trial, the frequency combination is generated randomly within the frequency span. The other basic parameters are the same as that in the first simulation. The target information to be estimated is set as R=5km, V=2m/s, A=0.5+0.8j.

**Figure 3:**
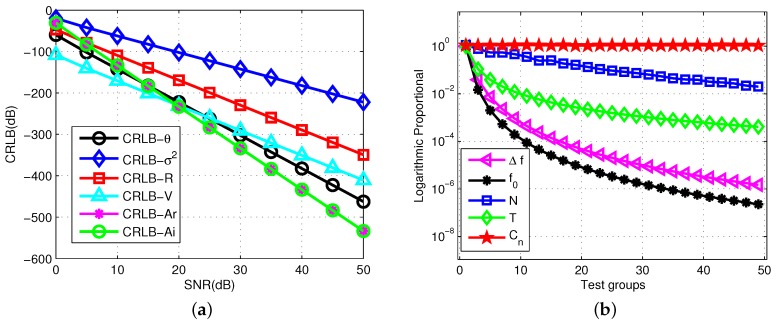
Comparison of CRLBs with changing SNR. (**a**) Monte Carlo Comparison of CRLBs; (**b**) Monte Carlo simulation of CRLB-θ with multiple variables.

The estimation performance of every $\mathrm{CRL}B_{i}$ is illustrated in Figure 3a. It shows the contribution of each separated value to the overall CRLB by changing the SNR. If the best estimation performance is synthesized, the parameters related to the single coefficient to be estimated can be adjusted appropriately.

To observe the trend of performance caused by various parameters more intuitively, a statistical analysis is presented in Figure 2b. The differences of the effects from various factors on the estimation performance are shown clearly. For each group of parameters, 500 independent Monte Carlo simulations are performed to obtain a statistical trend. The primary parameters of the variables are shown in Table 1.

${\left(\cdot \right)}^{\left(0\right)}$ represents the initial value for any variables; ${\left(\cdot \right)}^{\left(i\right)}$ represents the value in the $i^{th}$ group. When testing a variable, other variables are defined as the initial value. For the parameters of every group, the logarithmic proportional function is used to characterize the trend, that is $P\left(i\right)=\log\frac{\mathrm{var}{\left(\cdot \right)}^{\left(i\right)}}{\mathrm{var}{\left(\cdot \right)}^{\left(0\right)}}$. The testing variables are the frequency combination coefficient C, frequency number *N*, environment parameter $\sigma^{2}$, PRI *T*, frequency step Δf and initial carrier frequency $f_{0}$. As shown in the figure, the changes in Δf, $f_{0}$, and *N* exhibit corresponding effects on the range of the final CRLB. However, only Δf exhibits uniform variations; the other two variables exhibit differentiations under equidistant values. The effect of random combination is minimal when the other conditions remain unchanged.

The related parameters are divided broadly into the following categories.

Envelope of signal: When γ=0, $\mathrm{var}{\left(\cdot \right)}_{chirp}=\mathrm{var}{\left(\cdot \right)}_{rect}$. The value of γ has little effect. Because the value is sufficiently small, and the most of this coefficient concentrate in the items of *n* sequence with $f_{n}>>n>\gamma$, it implies that the envelope is not the dominant factor.

SNR, velocity, and amplitude: The real coefficient containing π, *c*, and $\sigma^{2}$ will directly affect the calculation results. The three items $N_{0}$, σ, $\left|a\right|$ are related to the SNR, velocity, and amplitude, respectively. The SNR can be obtained from the power and PSD, namely, $SNR=E/N_{0}$. Specifically, CRLB is proportional to σ, and inversely proportional to $N_{0}$.

Sparse frequency combination, frequency number *N*: When the value of *N* is fixed, the using signal bandwidth and the synthetic bandwidth are constant for each sparse frequency combination. Thus, the combination of frequencies does not have a significant effect. However, the maximum interval between two frequencies and the dispersion of every combination will impose higher requirements on the recovery method. Different methods involve particular constraints on the relationship between *N* and *M*, as well as special requirements for the rule of frequency hopping. We will not discuss this in detail; only the performance comparison will be emphasized herein.

Initial carrier frequency, pulse width, frequency step, and PRI: These parameters are determined by the signal, and are not changed by the environment and target scene. The trends of the three parameters is are synchronized. The value of $f_{0}$ is typically large, reaching MHz or even GHz, which is significantly larger than any other parameters. Therefore, it is the most significant factor. Conversely, *T* and $T_{w}$ are negligible owing to their small magnitudes.

### 6.3. CRLBs Using Different Estimators

**Figure 4:**
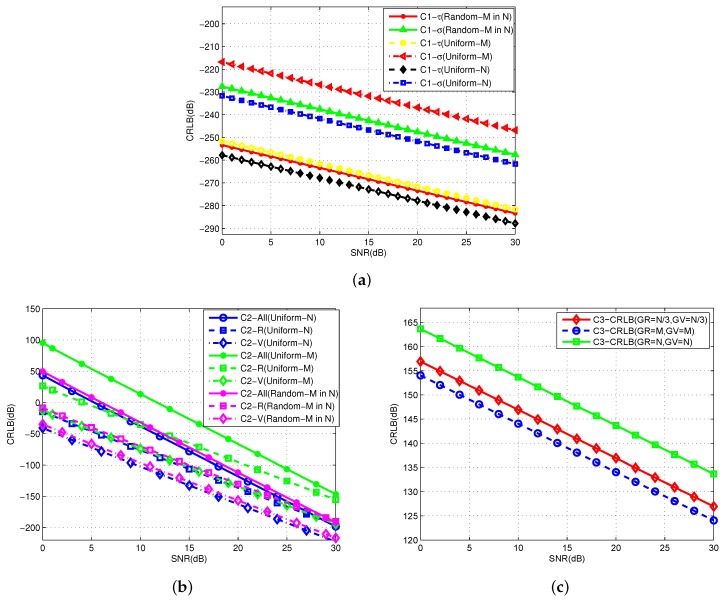
Comparison of CRLBs using different model. (**a**) CRLB using time delay and Doppler stretch estimator; (**b**) CRLB using multiple parameter estimator; (**c**) CRLB using the sparse based estimator.

This simulation uses the bound comparison produced by different estimators. The symbols C1, C2, and C3 are used to represent the time delay and Doppler stretch estimator, joint multiple parameter estimator, and sparse based estimator, respectively. The comparison results are shown in Figure 4.

The general parameters are set with $T_{w}=1\;\mathrm{ms}$, T=4ms, M=50, N=80, Δf=1kHz, $R_{0}=15\;\mathrm{km}$, $V_{0}=2\;m/s$, $A_{0}=-0.5+0.5j$, and the SNR is changed from 0 to 30 dB. In Figure 4a, the sampling rule includes the uniform *M* frequencies in *M* pulses, uniform *N* frequencies in *N* pulses, and random *M* sequences from *N* frequencies with *M* pulses. The time delay τ and Doppler stretch σ estimation are tested. In Figure 4b, the primary target information is measured: target range *R*, velocity *V*, and amplitude *A* under different sampling models. Jointly, the obtained overall CRLB C2-All is shown in the same figure. In Figure 4c, the sparse estimation is considered with the method based on grid partition. Therefore, it depends on the numbers of range grids GR and velocity grids GV. We use three groups of different grid numbers as an example.

As shown in the figure, the CRLB index is changed owing to the different samplings. In the uniform samplings, more samplings produce better estimation performance. However, the transmitting frequencies chosen randomly are somewhere in between. Therefore, we conclude from the statistical estimation, part of the estimation can be consistent with the theoretical value, while the estimation performance remains unchanged when the SNR is large.

### 6.4. RD Comparison between Different Methods

**Figure 5:**
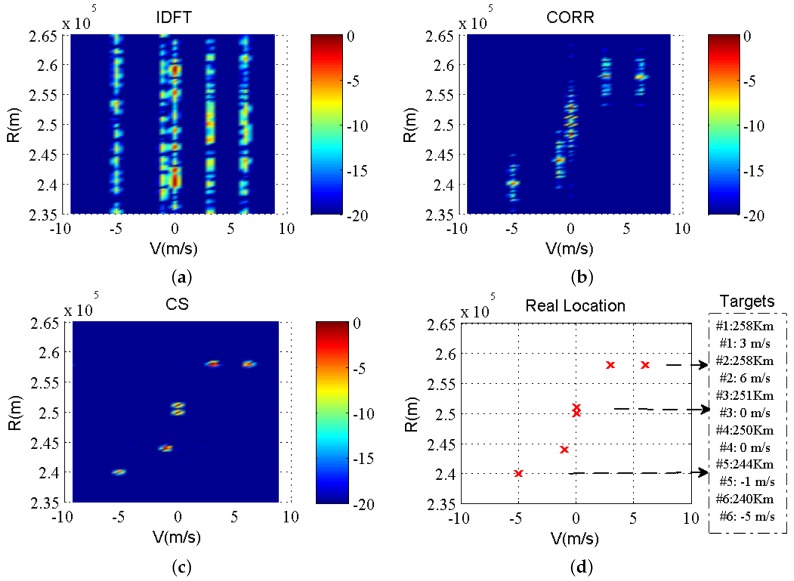
Target RD estimation with different method. (**a**) IDFT method; (**b**) Correlation function method; (**c**) compressive sensing method; (**d**) actual location of targets as a reference (The theoretical values of the actual range and velocity location are the discrete points in (**d**), which only provide references for estimation in (**a**–**c**)).

A practical example is presented to demonstrate the effectiveness of the target location using different estimation methods. We use the IDFT method, correlation function method [18], and CS method to estimate the range and velocity as a practical example. The contrasting figures illustrate the effect of resolution improvement and sidelobe suppression in the range domain. An SSF transmitting signal is designed using all available frequencies. The system parameters are set with carrier frequency $f_{0}=7.5\;\mathrm{MHz}$, sample frequency $f_{s}=1\;\mathrm{kHz}$, pulse width $T_{w}=1\;\mathrm{ms}$, PRI T=4ms, frequency span is 500kHz, pulse number M=250, step size Δf=1kHz. Six targets are considered with the parameter configuration as (#1) $R_{1}=258\;\mathrm{km}$, $V_{1}=3\;m/s$, (#2) $R_{2}=258\;\mathrm{km}$, $V_{2}=6\;m/s$, (#3) $R_{3}=251\;\mathrm{km}$, $V_{3}=0\;m/s$, (#4) $R_{4}=250\;\mathrm{km}$, $V_{4}=0\;m/s$, (#5) $R_{5}=244\;\mathrm{km}$, $V_{5}=-1\;m/s$, (#6) $R_{6}=240\;\mathrm{km}$, $V_{6}=-5\;m/s$. The SNR for every target is set to the same value 20dB. Thus, the frequency utilization rate is only 46%. We set multiple moving targets in the line-of-sight with the range and velocity values shown in Figure 5d.

Targets #3 and #4 are located at a close proximity with the same (zero) velocity. For the convenience of observation, the targets are shown with the same amplitude and SNR. Two-dimensional signal processing for the echo signals is performed from 50 burst pulses transmitted continuously. Processing methods are used to estimate the information of the targets. The comparison results of the three methods are shown in Figure 5a–c.

It is apparent that the IDFT method cannot determine the location of the targets. The range and velocity information of the targets can be obtained by the correlation function method. However, owing to the effect of the high-range sidelobe level, this method cannot distinguish the exact location of the targets. If we do not consider the ambiguity caused by the radial velocity, and only focus on the sidelobe suppression in the range domain, the CS method yields a better performance in improving the precision of the range measurement. The selected observation range of the axis is −20 to 0 dB. As shown, the sidelobe below −20 dB have little effect on the targets; additionally, it is easy to distinguish two closely spaced targets.

**Figure 6:**
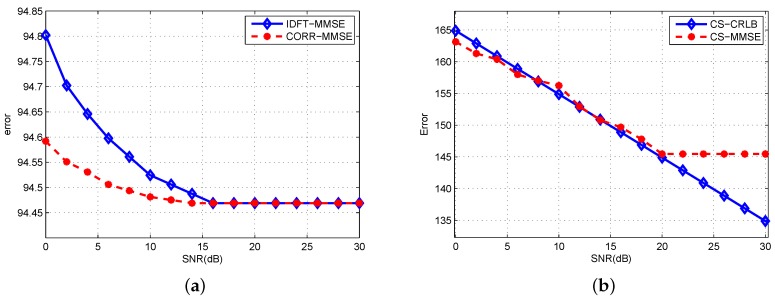
Monte Carlo tests of target estimation. (**a**) MMSE of IDFT and CORR method; (**b**) CRLB and MMSE of CS method.

Next we will give a statistical result for IDFT, CORR and CS method. In Figure 6, by adding the range, velocity and the amplitude estimated error together, the statistical values of the minimum mean square error (MMSE) are tested by Monte Carlo simulations. The times of Monte Carlo is 500, SNR from 0 to 30 dB. For CS, the traditional orthogonal matching pursuit method is chosen here. Three different methods are drawn separately because of the difference in error value level. Figure 6a illustrates the MMSE of IDFT and CORR method. The results of comparison between CRLB and MMSE of CS method are shown in Figure 6b.

As shown in the figure, with the increase of SNR, the trend of MMSE is decreased before stable in a certain value until it reaches a large SNR. CORR method has the fastest speed to achieve stable accuracy, and CS method has the best robustness in this test. The results of theoretical CRLB and MMSE are consistent with CS method. When SNR exceeds 20 dB, the limitation of grids will lead to a same error without further reduction.

## 7. Conclusions

In this paper, the CRLB performance by different representations was analyzed under the conversion of multiple equivalent models. Several theoretical derivations were investigated for different estimation methods; they will serve as reference and guidance for the design of transmission waveforms in the front end and the estimation of signals in the back end. With these specific expressions, the estimation performances for different parameter combinations could be determined directly. With increasing Δf, $f_{0}$, *N*, and *T*, CRLB performance was improved. Additionally, environmental noise as an extremely important factor should be considered. Meanwhile, regarding the determined frequency width, the effect of frequency combination based on statistics was much weaker for this type of signal. The theoretical results are beneficial for target estimation and detection by introducing an SSF signal into a radar system.

## Figures and Tables

**Table 1:** Parameters setting for multiple groups of SSF signal.

Variables	Initial Value	the Value of $i^{\mathrm{th}}$ Groups	Value Range
$\sigma^{2}$	${\left(\sigma^{2}\right)}^{\left(0\right)}=1$	${\left(\sigma^{2}\right)}^{\left(i\right)}=10^{2(i/20)}$	i=[1,50]
Δf (kHz)	$\Delta f^{\left(0\right)}=1$	$\Delta f^{\left(i\right)}=\Delta f^{\left(0\right)}+\left(10i-1\right)$	i=[1,50]
$f_{0}$ (MHz)	${f_{0}}^{\left(0\right)}=1$	${f_{0}}^{\left(i\right)}={f_{0}}^{\left(0\right)}+\left(i-1\right)$	i=[1,50]
*N*	$N^{\left(0\right)}=40$	$N^{\left(i\right)}=N^{\left(0\right)}+\left(i-1\right)$	i=[1,50]
*T*(ms)	$T^{\left(0\right)}=1$	$T^{\left(i\right)}=T^{\left(0\right)}+\left(i-1\right)$	i=[1,50]
C	Random	Random	$C_{n}\in \left[1,M\right]$
